# Derivation and validation of a nomogram for predicting nonventilator hospital-acquired pneumonia among older hospitalized patients

**DOI:** 10.1186/s12890-022-01941-z

**Published:** 2022-04-15

**Authors:** Zhihui Chen, Ziqin Xu, Hongmei Wu, Shengchun Gao, Haihong Wang, Jiaru Jiang, Xiuyang Li, Le Chen

**Affiliations:** 1 Department of Infection Control, Wenzhou People’s Hospital, 57 Canghou Road, Wenzhou, China; 2 Department of Epidemiology & Statistics, and Center for Clinical Big Data and Statistics, Second Affiliated Hospital, Zhejiang University College of Medicine, 866 Yuhangtang Road, Hangzhou, 310058 China

**Keywords:** Hospital-acquired pneumonia, Aspiration pneumonia, Infection prevention, Nomogram, Prediction model

## Abstract

**Background:**

Currently, there is no effective tool for predicting the risk of nonventilator hospital-acquired pneumonia (NV-HAP) in older hospitalized patients. The current study aimed to develop and validate a simple nomogram and a dynamic web-based calculator for predicting the risk of NV-HAP among older hospitalized patients.

**Methods:**

A retrospective evaluation was conducted on 15,420 consecutive older hospitalized patients admitted to a tertiary hospital in China between September 2017 and June 2020. The patients were randomly divided into training (n = 10,796) and validation (n = 4624) cohorts at a ratio of 7:3. Predictors of NV-HAP were screened using the least absolute shrinkage and selection operator method and multivariate logistic regression. The identified predictors were integrated to construct a nomogram using R software. Furthermore, the optimum cut-off value for the clinical application of the model was calculated using the Youden index. The concordance index (C-index), GiViTI calibration belts, and decision curve were analysed to validate the discrimination, calibration, and clinical utility of the model, respectively. Finally, a dynamic web-based calculator was developed to facilitate utilization of the nomogram.

**Results:**

Predictors included in the nomogram were the Charlson comorbidity index, NRS-2002, enteral tube feeding, Barthel Index, use of sedatives, use of NSAIDs, use of inhaled steroids, and "time at risk". The C-index of the nomogram for the training and validation cohorts was 0.813 and 0.821, respectively. The 95% CI region of the GiViTI calibration belt in the training (*P* = 0.694) and validation (*P* = 0.614) cohorts did not cross the diagonal bisector line, suggesting that the prediction model had good discrimination and calibration. Furthermore, the optimal cut-off values for the training and validation cohorts were 1.58 and 1.74%, respectively. Analysis of the decision curve showed that the nomogram had good clinical value when the threshold likelihood was between 0 and 49%.

**Conclusion:**

The developed nomogram can be used to predict the risk of NV-HAP among older hospitalized patients. It can, therefore, help healthcare providers initiate targeted medical interventions in a timely manner for high-risk groups.

**Supplementary Information:**

The online version contains supplementary material available at 10.1186/s12890-022-01941-z.

## Introduction

Hospital-acquired pneumonia (HAP) is a common healthcare-associated infection (HAI) that affects approximately 1% of all hospitalized patients [1–3]. Nonventilator hospital-acquired pneumonia (NV-HAP) and ventilator-associated pneumonia (VAP) are the two subtypes of HAP [4]. The former (NV-HAP) accounts for more than 60% of HAP cases [2]. Nonventilator hospital-acquired pneumonia has been linked to longer hospital stays, extensive use of intensive care services, higher medical costs, and a high risk of hospital mortality [5, 6]. Several unmodifiable and modifiable risk factors, such as age (> 65 years), surgery, enteral feedings, lack of mobility, malnutrition, high blood glucose, inhibition of gastric acid, and central nervous system depressants, that predict the occurrence of NV-HAP have been previously profiled [7]. Evidence from a previous systematic review indicates that interventions such as improved oral hygiene, dysphagia screening, and early mobilization targeting potential modifiable risk factors might reduce the risk of NV-HAP [8]. Although NV-HAP is preventable and controllable, the widespread nature of the disease among clinical departments necessitates the search for various preventive interventions targeted at inpatients [9, 10]. The incidence of NV-HAP increases with age, and it has been found that older patients have a higher mortality rate and a worse prognosis than younger patients [9, 11]. Therefore, identifying populations at a high risk of NV-HAP, especially in older hospitalized patients, is crucial, as it will allow early initiation of interventions to prevent the occurrence of NV-HAP. Furthermore, early and accurate identification of high-risk groups will lead to the design of effective policies and preventive measures for NV-HAP.

To date, few risk prediction tools for NV-HAP in older hospitalized patients have been developed. A nomogram is a visualization method for complex mathematical models that combines multiple risk factors. This method provides accurate and individualized risk estimates for patients and presents them intuitively [12, 13]. Therefore, the present study aimed to develop and validate a simple nomogram and a dynamic web-based calculator for predicting NV-HAP risk among older hospitalized patients.

## Methods

### Study design and participants

The current study adopted a retrospective cohort study design. A total of 15,420 consecutive older hospitalized patients admitted to Wenzhou People's Hospital (a 1500-bed teaching hospital in Zhejiang, China) between September 2017 and June 2020 were recruited. Patients with a stay length of < 48 h and those who had received mechanical ventilation during hospitalization were excluded from the study. In the case of repeated admissions for a patient, only the first admission was considered. The current study was approved by the Ethics Review Committee of Wenzhou People's Hospital (IRB no: WRY2018070). The same committee waived the requirement for informed consent due to the retrospective nature of the study.

### Candidate predictors

The following candidate predictors were collected: demographic data (age, sex), Nutritional Risk Screening 2002 (NRS-2002), Barthel Index, Morse Fall Scale, other nosocomial infections, season of admission, Charlson comorbidity index (CCI), and admission category. Furthermore, the patient's personal history (drinking status, smoking status), comorbidities (chronic obstructive pulmonary disease (COPD), swallow disability, stroke, diabetes mellitus, peptic ulcer disease, moderate or severe renal disease, liver disease, congestive heart failure, and solid tumor), clinical procedures (central venous catheter, indwelling urinary catheter, surgery, parenteral nutrition, and enteral tube feeding), in-hospital medications (antacids, sedatives, nonsteroidal anti-inflammatory drugs (NSAIDs), systemic steroids, inhaled steroids, and anticoagulant) and laboratory values (blood urea nitrogen, albumin, C-reactive protein, lymphocytes, white blood cells, and hemoglobin) were collected. The burden of comorbid diseases was described using the CCI [14]. The NRS-2002 [15], Barthel Index [16], and Morse Fall Scale [17] were used to assess the nutritional status, performance in activities of daily living, and risk of falling among inpatients, respectively.

To determine the effect of time on NV-HAP, the "time at risk" was also included as a potential predictor. For patients with NV-HAP, "time at risk" was calculated as the NV-HAP diagnosis date minus the admission date. For patients without NV-HAP, the "time at risk" was determined as the total length of stay. The variable "time at risk" was categorized into three subgroups based on tertile points, ≤ 6, 7–11, and ≥ 12 days. Laboratory data were collected from the first-time examinations at admission. During the "time at risk" period, clinical procedures, other nosocomial infections, and in-hospital medications were also collected.

### Outcome

The data on NV-HAP were drawn from the Xinglin system, which is a real-time nosocomial infection surveillance system [18]. The system can collect data from multiple hospital databases [including Hospital Information System (HIS), Laboratory Information System (LIS), and Remote Installation Service systems (RIS)] in real time. In addition, the system can report automatically screened infection warnings, such as information on clinical signs, positive bacterial culture, and elevated biochemical and inflammatory indexes.

For nosocomial infection cases, clinicians made an initial diagnosis, which was then confirmed by a senior infection control practitioner. In case of a discrepant diagnosis between the two sides of the diagnosis, a joint discussion was held to reach a consensus. NV-HAP was defined according to the 2018 version of the Chinese guidelines for diagnosing and treating hospital-acquired pneumonia and ventilator-associated pneumonia in adults [19]. The definition was also consistent with the guidelines issued by the American Thoracic Society [20].

### Feature selection and construction of a prediction model

The least absolute shrinkage and selection operator (LASSO) method was used to select optimal predictive features from the aforementioned 38 candidate predictors in the training cohort [21]. The LASSO method can reduce highly dimensional data and provides a better-fitting model. The optimal value of the penalty parameter λ was determined through a tenfold cross validation. Variables with nonzero coefficients in the fitted LASSO model were considered significant predictors. To obtain an integrated nomogram, a stepwise feature selection algorithm with the Akaike information criterion (AIC) and multivariate logistic regression model were used to filter the predictors [22]. The regression coefficients of the selected independent variables were used to construct a simple nomogram for predicting NV-HAP risk in older hospitalized patients.

### Evaluating the model's performance

The performance of the model in predicting NV-HAP risk was expressed in terms of discrimination and calibration. Discrimination of the prediction model refers to the ability to distinguish between individuals developing and not developing the outcome (NV-HAP) [23]. The concordance index was used to validate the nomogram's discrimination ability (C-index; equal to the area under the receiver operating curve). The value of the C-index varied from 0.5 to 1.0, with values > 0.75 indicating relatively good discrimination.

The calibration of a prediction model refers to testing the agreement between the predicted and actual observed risk [23]. The GiViTI calibration belts were plotted to calibrate the nomogram [24]. The deviation between the predicted and observed probabilities was detected using the 0.95 confidence band of the calibration curve and calibration test. The 95% CI did not cross the bisector, which indicated a statistically significant deviation from the predicted probabilities. A *P* value > 0.05 in the calibration test demonstrated that there was no evidence of poor fit in the nomogram.

### Clinical usage

Decision curve analysis (DCA) was performed to assess the clinical utility of the developed nomogram [25]. Decision curve analysis (DCA) is a tool for assessing the potential benefits of a risk prediction model once applied in clinical practice. It is calculated as shown in the formulae below.

$${\text{Net}}\;{\text{benefit}} = {\text{true}}\;{\text{positive}}\;{\text{rate}} - {\text{false}}\;{\text{positive}}\;{\text{rate}} \times \frac{{P_{{\text{t}}} }}{{1 - P_{{\text{t}}} }}$$

where *P*t represents the threshold probability at which the expected benefit of intervention-all-patients is equal to the expected benefit of intervention-none.

In addition, the highest Youden index was used to obtain the best cut-off value for clinical application (sensitivity + specificity − 1).

### Statistical analysis

The present study was reported as per the Transparent Reporting of a multivariable prediction model for Individual Prognosis or Diagnosis (TRIPOD) statement [26]. All the included patients were randomly divided into the training (n = 10,796) and validation (n = 4624) cohorts at a ratio of 7:3 [27, 28]. The training cohort was used for model training and estimation of model parameters, whereas the validation cohort was used to test the model and evaluate its benefits. Nonnormally distributed continuous data were analyzed with the Mann–Whitney U test and expressed as medians (inter-quartile range, *IQR*). Categorical variables were expressed as numbers (%) and compared using the chi-square (and Fisher's exact) test. Patients with missing data were excluded from this study because the proportion of cases with missing data was minimal (< 5%), and it was considered to be missing at random [29, 30]. All data were statistically analyzed using R software (version 3.6.1; https://www.R-project.org). A two-tailed *P* value of ≤ 0.05 was considered to be statistically significant.

## Results

### Characteristics of the study cohort

A total of 15,420 participants were included in the final analysis (Additional file 1: Fig. S1). A total of 10,796 and 4624 participants belonged to the training cohort and validation cohort, respectively. In the training cohort, 153 cases were NV-HAP, and 10,643 cases were non-NV-HAP. In the validation cohort, 72 cases were NV-HAP, and 4552 cases were non-NV-HAP. The percentage of patients with NV-HAP in the training and validation cohorts was 1.4% and 1.6%, respectively, and the difference was not statistically significant (*P* = 0.507). The baseline characteristics of patients in the training and validation cohorts are presented in Table 1.

**Table 1:** Baseline characteristics of the study population

Characteristic	Training Cohort (n = 10,796)			Validation Cohort (n = 4624)		
	NV-HAP (n = 153)	Non-NV-HAP (n = 10,643)	*P*	NV-HAP (n = 72)	Non-NV-HAP (n = 4552)	*P*
Age(years), median (IQR^†^)	79 (12)	73 (12)	< 0.001	78 (9)	73 (12)	< 0.001
Male, n (%)	90 (58.8)	5598 (52.6)	0.126	34 (56.7)	2351 (52.2)	0.496
Drinking status, n (%)			0.400			0.406
Never drinker	129 (84.3)	8732 (82.0)		61 (84.7)	3769 (82.8)	
Current drinker	13 (8.5)	1262 (11.9)		5 (6.9)	509 (11.2)	
Former drinker	11 (7.2)	649 (6.1)		6 (8.3)	274 (6.0)	
Smoking status, n (%)			0.597			0.417
Never smoker	124 (81.0)	8535 (80.2)		58 (80.6)	3646 (80.1)	
Current smoker	13 (8.5)	1142 (10.7)		5 (6.9)	486 (10.7)	
Former smoker	11 (7.2)	966 (9.1)		9 (12.5)	420 (9.2)	
Comorbidities, n (%)						
COPD	5 (3.3)	391 (3.7)	0.791	2 (2.8)	173 (3.8)	0.652
Swallow disability	5 (3.3)	57 (0.5)	< 0.001	0 (0.0)	29 (0.6)	0.497
Stroke	67 (43.8)	3027 (28.4)	< 0.001	39 (54.2)	1279 (28.1)	< 0.001
Diabetes mellitus	49 (32.0)	2963 (27.8)	0.252	28 (38.9)	1288 (28.3)	0.048
Peptic ulcer disease	6 (3.9)	352 (3.3)	0.674	1 (1.4)	191 (4.2)	0.236
Moderate or severe renal disease	17 (11.1)	667 (6.3)	0.015	7 (9.7)	280 (6.2)	0.213
Liver disease	22 (14.4)	2036 (19.1)	0.137	16 (22.2)	927 (20.4)	0.698
Congestive heart failure	5 (3.3)	129 (1.2)	0.023	6 (8.3)	79 (1.7)	< 0.001
Solid tumour	36 (23.5)	1400 (13.2)	< 0.001	17 (23.6)	572 (12.6)	0.005
CCI (points), median (IQR)	6(3)	4 (3)	< 0.001	6 (2)	4(2)	< 0.001
Time at risk(days)			0.164			0.132
≤ 6	48 (31.4)	3741 (35.1)		21 (29.2)	1672 (36.7)	
7–11	49 (32.0)	3758 (35.3)		22 (30.6)	1534 (33.7)	
≥ 12	56 (36.6)	3144 (29.5)		29 (40.3)	1346 (29.6)	
Admission category, n (%)			0.003			0.413
Internal medicine	90 (58.8)	6856 (64.4)		51 (70.8)	2934 (64.5)	
Surgery	55 (35.9)	2857 (26.8)		20 (27.8)	1239 (27.2)	
Gynaecology	4 (2.6)	219 (2.1)		1 (1.4)	110 (2.4)	
Emergency department	1 (0.7)	477 (4.5)		0 (0.0)	190 (4.2)	
ICU	3 (2.0)	59 (0.6)		0 (0.0)	24 (0.5)	
Others	0 (0.0)	175 (1.6)		0 (0.0)	55 (1.2)	
Clinical procedure, n (%)						
Central venous catheter	30 (19.6)	526 (4.9)	< 0.001	20 (27.8)	249 (5.5)	< 0.001
Indwelling urinary catheter	49 (32.0)	1652 (15.5)	< 0.001	22 (30.6)	735 (16.1)	0.001
Surgery	32 (20.9)	1792 (16.8)	0.181	13 (18.1)	764 (16.8)	0.775
Parenteral nutrition	18 (11.8)	472 (4.4)	< 0.001	10 (13.9)	209 (4.6)	< 0.001
Enteral tube feeding	57 (37.3)	1165 (10.9)	< 0.001	28 (38.9)	524 (11.5)	< 0.001
NRS-2002(points), median (IQR)	2 (2)	1 (2)	< 0.001	2 (2)	1(2)	< 0.001
Barthel Index, n (%)			< 0.001			< 0.001
Independent	51 (33.3)	7338 (68.9)		22 (30.6)	3081 (67.7)	
Slight dependency	19 (12.4)	1541 (14.5)		12 (16.7)	638 (14.0)	
Moderate dependency	20 (13.1)	894 (8.4)		6 (8.3)	447 (9.8)	
Severe dependency	31 (20.3)	528 (5.0)		16 (22.2)	241 (5.3)	
Total dependency	32 (20.9)	342 (3.2)		16 (22.2)	145 (3.2)	
Morse Fall Scale, n (%)			< 0.001			0.046
No risk	19 (12.4)	1945 (18.3)		7 (9.7)	803 (17.6)	
Low risk	76 (49.7)	6555 (61.6)		44 (61.1)	2872 (63.1)	
High risk	58 (37.9)	2143 (20.1)		21 (29.2)	877 (19.3)	
Other nosocomial infections, n (%)	4 (2.6)	282 (2.6)	0.978	3 (4.2)	110 (2.4)	0.340
Season of admission, n (%)			0.437			0.141
Spring	42 (27.5)	2684 (25.2)		19 (26.4)	1208 (26.5)	
Summer	33 (21.6)	2813 (26.4)		11 (15.3)	1208 (26.5)	
Fall	38 (24.8)	2245 (21.1)		20 (27.8)	970 (21.3)	
Winter	40 (26.1)	2901 (27.3)		22 (30.6)	1166 (25.6)	
In-hospital medications, n (%)						
Antacids	119 (77.8)	7362 (69.2)	0.022	55 (76.4)	3099 (68.1)	0.133
Sedatives	40 (26.1)	1521 (14.3)	< 0.001	16 (22.2)	650 (14.3)	0.057
NSAIDs	45 (29.4)	1350 (12.7)	< 0.001	14 (19.4)	565 (12.4)	0.074
Systemic steroids	23 (15.0)	1893 (17.8)	0.376	18 (25.0)	832 (18.3)	0.144
Inhaled steroids	36 (23.5)	1140 (10.7)	< 0.001	18 (25.0)	513 (11.3)	< 0.001
Anticoagulant	50 (32.7)	1685 (15.8)	< 0.001	21 (29.2)	745 (16.4)	0.004
Laboratory values, median (IQR)						
BUN, mmol/L	11.0 (9.6)	10.8 (7)	0.584	9.4 (7.7)	10.8 (6.9)	0.079
ALB,g/L	36.0 (7)	38.6 (6.3)	< 0.001	35.3 (6.4)	38.5 (6.1)	< 0.001
CRP, mg/L	12.5 (31.9)	3.4 (17.6)	< 0.001	14.2 (38.5)	3.7 (18.4)	< 0.001
LY,10^9^ /L	1.2 (0.7)	1.4 (0.9)	< 0.001	1.2 (0.8)	1.4 (0.8)	0.003
WBC,10^9^ /L	7.7 (3.7)	6.4 (2.9)	< 0.001	7.4 (3.3)	6.4 (2.9)	0.006
Hb,g/L	118.0 (31.0)	126.0 (23.0)	< 0.001	111.5 (32.4)	126.0 (22.0)	< 0.001

*NRS* nutritional risk screening; *IQR* inter-quartile range; *COPD* chronic obstructive pulmonary disease; *CCI* Charlson comorbidity index; *ICU* intensive care unit; *NSAIDs* non-steroidal anti-inflammatory drug; *NV-HAP* non-ventilator-associated hospital-acquired pneumonia; *BUN* blood urea nitrogen; *ALB* albumin; *CRP* C-reaction Protein; *LY* lymphocyte; *WBC* white blood cell; *Hb* hemoglobin

^†^IQR means the distance between the first quartile and the third quartile

### Feature selection

**Fig. 1:**
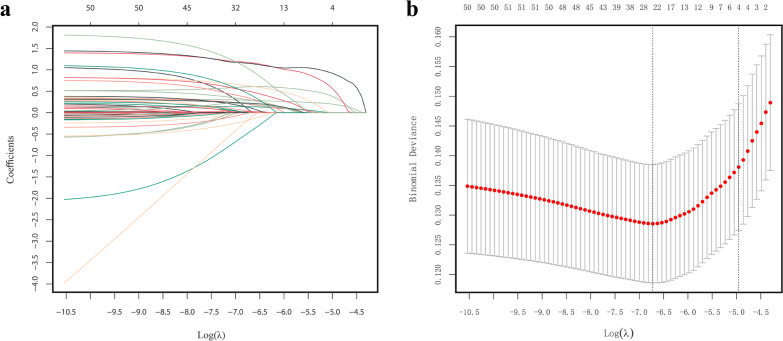
Variable selection using the LASSO binary logistic regression model. **a** Profiles of the LASSO coefficients for the 38 candidate variables. **b** Optimal penalization coefficient (λ) selection in the LASSO model using tenfold cross-validation via minimum criteria. Note: the left vertical line represents the minimum error, and the right vertical line represents the one standard error of the minimum criteria (1-SE criterion). *LASSO* least absolute shrinkage and selection operator

Among the 38 variables collected, 17 potential predictors were identified in the training cohort (Fig. 1a, b). They included the Charlson comorbidity index, Hb, NRS-2002, stroke, admission category, central venous catheter, indwelling urinary catheter, surgery, enteral tube feeding, Barthel Index, Morse Fall Scale, other nosocomial infections, use of sedatives, use of NSAIDs, use of inhaled steroids, use of anticoagulant, and "time at risk". The predictors showed nonzero coefficients in the LASSO regression model.

### Prediction model development

**Fig. 2:**
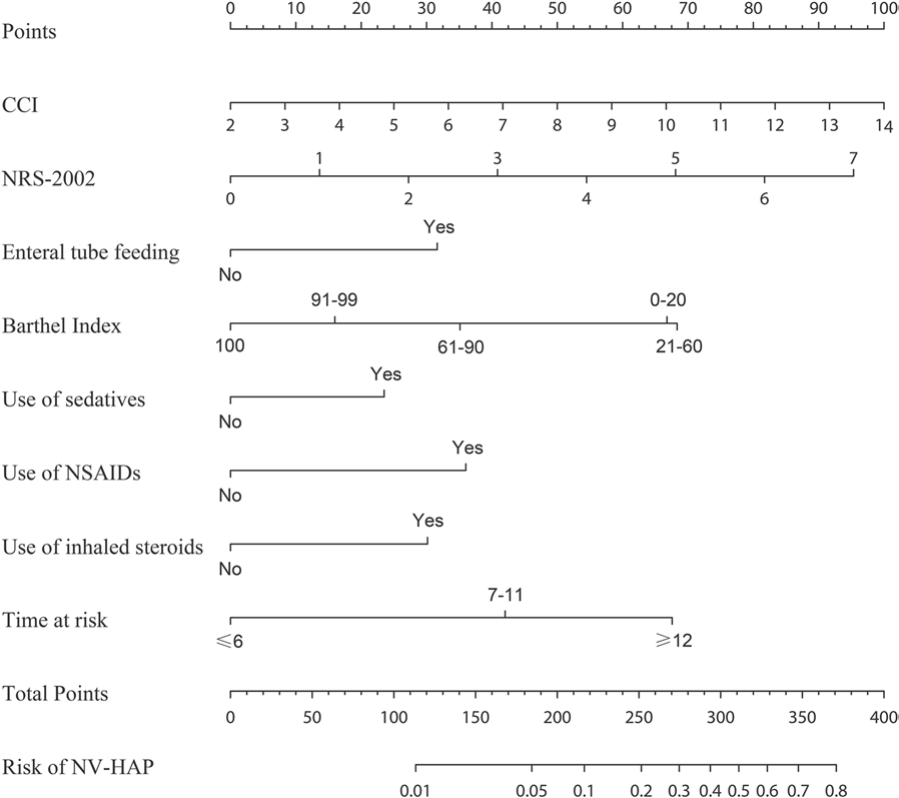
The nomogram for predicting the risk of NV-HAP in older hospitalized patients. *Note* The NV-HAP risk nomogram was developed with the predictors including CCI, NRS-2002, enteral tube feeding, Barthel Index, use of sedatives, use of NSAIDs, use of inhaled steroids, and “time at risk”. *NV-HAP* nonventilator-associated hospital-acquired pneumonia; *CCI* Charlson comorbidity index; *NRS* nutritional risk screening

To further identify independent predictors of NV-HAP, a multivariable logistic regression model with a two-way stepwise strategy was adopted. Finally, the model including CCI, NRS-2002, Enteral tube feeding, Barthel Index, use of sedatives, use of NSAIDs, use of inhaled steroids, and "time at risk" had the lowest AIC, hence the best goodness of fit (Table 2). Therefore, we used these eight independent predictors to construct a novel nomogram model for predicting the probability of NV-HAP (Fig. 2).

**Table 2:** Logistic analysis of each factor's ability in predicting the risk of NV-HAP

Intercept and variable	Prediction model		
	β	Odds ratio (95%CI)	*P*-value
Intercept	-7.617	0(0.000–0.001)	< 0.001
Charlson comorbidity index (point)	0.194	1.214(1.104–1.332)	< 0.001
NRS-2002	0.316	1.372(1.209–1.553)	< 0.001
Enteral tube feeding			
No	Reference		
Yes	0.735	2.085(1.369–3.136)	< 0.001
Barthel Index			
Independent	Reference		
Slight dependency	0.371	1.449(0.826–2.44)	0.177
Moderate dependency	0.816	2.261(1.285–3.835)	0.003
Severe dependency	1.588	4.894(2.937–8.028)	< 0.001
Total dependency	1.552	4.72(2.639–8.327)	< 0.001
Use of sedatives			
No	Reference		
Yes	0.546	1.727(1.145–2.555)	0.008
Use of NSAIDs			
No	Reference		
Yes	0.837	2.309(1.558–3.37)	< 0.001
Use of inhaled steroids			
No	Reference		
Yes	0.701	2.015(1.31–3.028)	0.001
Time at risk(days)			
≤ 6	Reference		
7–11	0.976	2.655(1.714–4.131)	< 0.001
≥ 12	1.570	4.808(3.087–7.555)	< 0.001

*NV-HAP* non-ventilator-associated hospital-acquired pneumonia; *NRS* nutritional risk screening; *NSAIDs* non-steroidal anti-inflammatory drugs; *CI* confidence interval

Meanwhile, the R package shiny was used to create a visual and operational dynamic web-based calculator for the developed model. The user can conveniently obtain the prediction probability of NV-HAP by entering or selecting a variable in the graphical user interface (https://predction.shinyapps.io/DynNomapp/). For instance, using the dynamic nomogram for NV-HAP, the probability of NV-HAP in an older hospitalized patient with CCI = 8, NRS-2002 = 3, no enteral tube feeding, Barthel Index = 95 (slight dependency), use of sedatives, use of NSAIDs, use of inhaled steroids, and "time at risk" = 7 days was estimated to be 15.6% (Additional file 1: Fig. S2).

### Prediction model validation

**Fig. 3:**
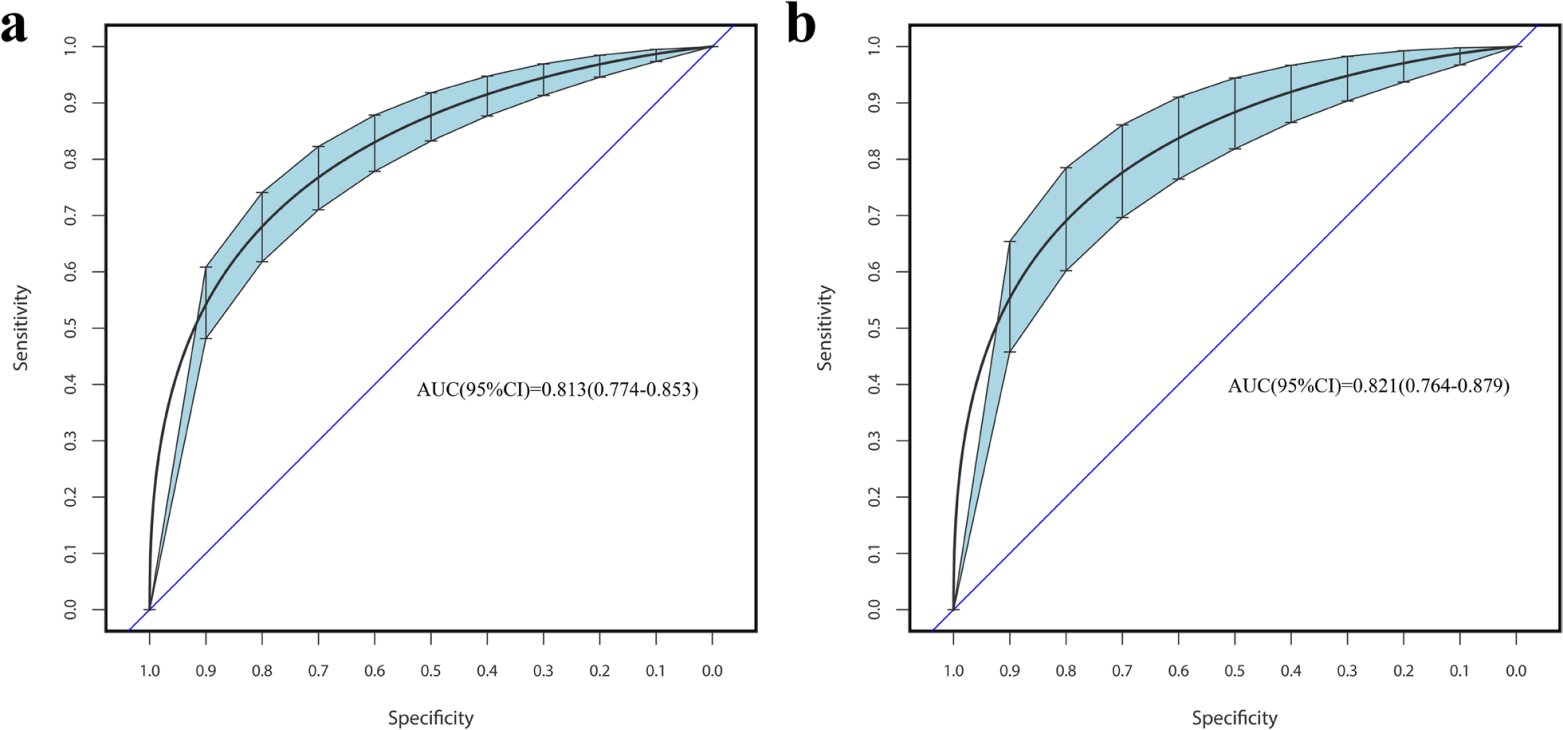
ROC curves of the nomogram. **a** The training cohort. **b** Validation Cohort. *Note* the x-axis represents the false-positive rate of the risk prediction. The y-axis indicates the true-positive rate of the risk prediction. *ROC* receiver operating characteristic; *AUC* area under the curve

The C-index of the nomogram in the training cohort was 0.813 (95% confidence interval: 0.774–0.853) (Fig. 3a), whereas that in the validation cohort was 0.821 (95% confidence interval: 0.764–0.879) (Fig. 3b). These results suggested that the developed prediction model had good discrimination.

**Fig. 4:**
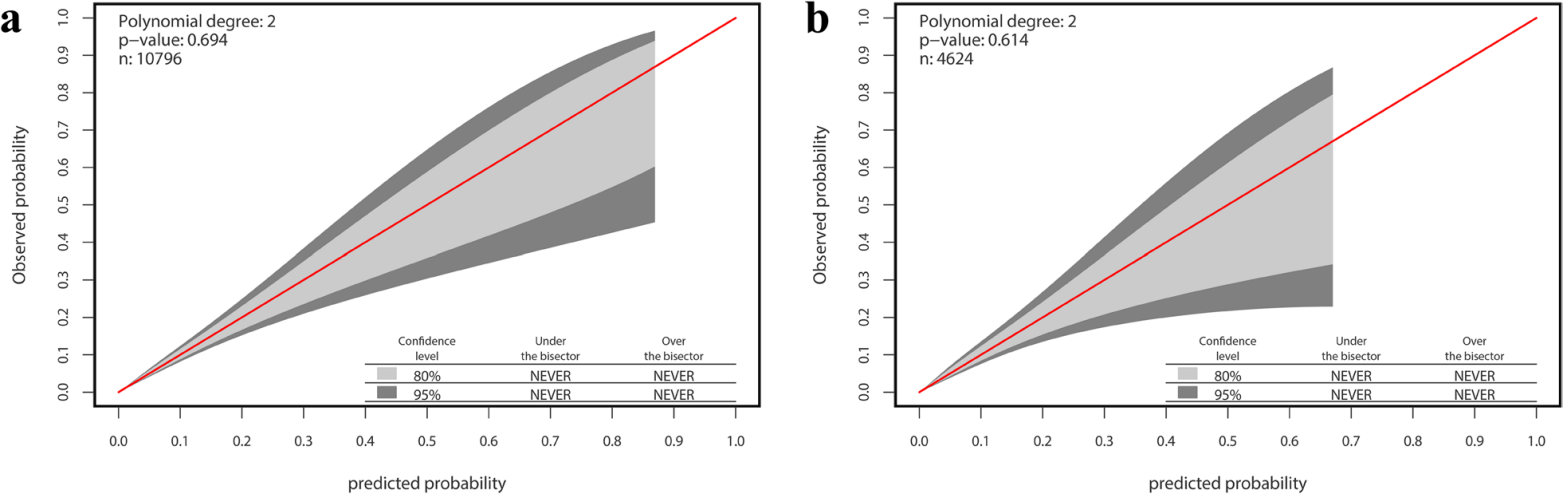
The GiViTI calibration belt for the nomogram. **a** The training cohort. **b** Validation Cohort. *Note* The 80% CI and 95% CI calibration belt are plotted in light and dark gray, respectively. The red diagonal line is the reference line indicating perfect calibration

The 95% CI region of the GiViTI calibration belt in the training and validation cohorts did not cross the diagonal bisector line (*P* = 0.694, *P* = 0.614; respectively) (Fig. 4a, b). This suggested that the developed prediction model had strong concordance performance in the two data sets.

### Determination of optimal cut-off values for the nomogram

At the maximum Youden index, the optimal cut-off values in the training and validation cohorts were 1.58 and 1.74%, respectively. The developed model was applied to predict NV-HAP in the studied cohorts (Additional file 1: Table S1). The following results were obtained in the training cohort: specificity (81.94%), sensitivity (69.93%), accuracy (81.77%), positive predictive value (PPV) (5.27%), negative predictive value (NPV) (99.48%), positive likelihood ratio (PLR) (3.87%), negative likelihood ratio (NLR) (0.37%), and diagnostic odds ratio (DOR) (10.55%). For the validation cohort, the following results were obtained: specificity (82.36%), sensitivity (70.83%), accuracy (82.18%), positive predictive value (PPV) (5.97%), negative predictive value (NPV) (99.44%), positive likelihood ratio (PLR) (4.02%), negative likelihood ratio (NLR) (3.54%), and diagnostic odds ratio (DOR) (11.34%).

### Clinical usefulness of the nomogram

**Fig. 5:**
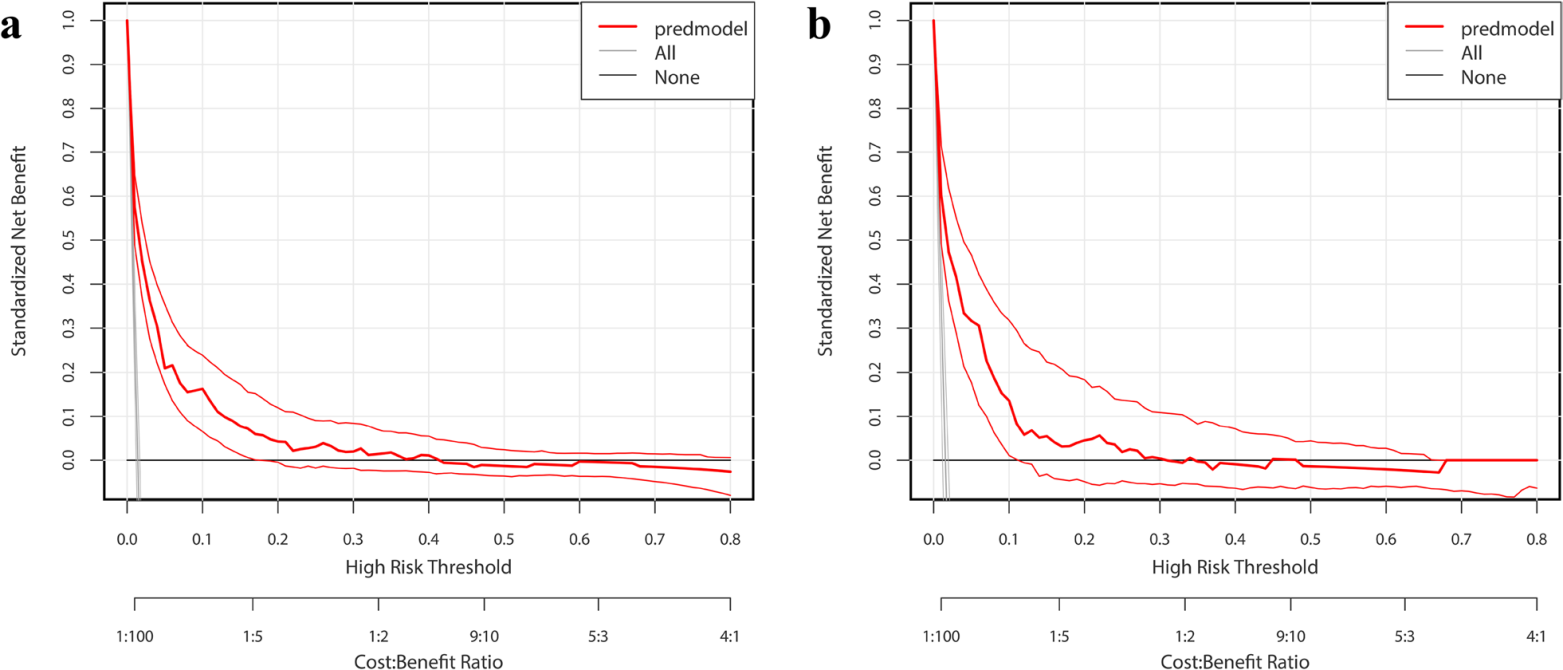
Decision curve analysis for the nomogram. **a** The training cohort. **b** Validation Cohort. *Note* The y-axis represents the standardized net benefit. The thick red solid line is the nomogram to predict NV-HAP risk. The thin red solid line represents the 95% credible interval. The black solid line represents the assumption that all patients had no NV-HAP. The gray solid line represented the assumption that all patients had NV-HAP

The results of the decision curve analysis of the risk nomogram for the training and validation cohorts are shown in Fig. 5a, b, respectively. At a likelihood range of between 0 and 49%, the nomogram showed better performance in predicting NV-HAP risk compared to intervention-all-patients or intervention-none strategies. For example, in the training cohort, the standardized net benefit was approximately 60% at the 1.58% probability threshold.

## Discussion

Nonventilator hospital-acquired pneumonia (NV-HAP) is a major safety issue among hospitalized patients and leads to high treatment costs, mortality rates, and longer hospital stays. Therefore, it is important to develop risk prediction models that can accurately identify high-risk groups to facilitate the early implementation of preventive interventions. Researchers have developed tools for measuring the risk of NV-HAP. For instance, Evans [31] developed an NV-HAP risk assessment tool based on risk factors reported by previous studies. In his assessment tool, each risk was weighted depending on the specific nature of the research and the number of authors who proposed the risk factors rather than using rigorous mathematical procedures. In their study, they conducted a hypothetical risk assessment, and thus further evaluation of the proposed risk factors and the weighting method is required. In a separate study, Wolfensberger et al. [32] developed and validated an automated classification algorithm based on radiological procedure (X-ray or CT scan) criteria to distinguish patients 'not at risk' from patients 'at risk' of developing NV-HAP. However, the primary aim of this automated classification algorithm was to reduce the manual surveillance workload rather than predicting the development of NV-HAP. Currently, there is no effective tool for assessing the risk of NV-HAP in patients, especially older hospitalized patients. Therefore, the current study aimed to develop and validate a simple-to-use nomogram and to establish an online calculator for the early prediction of NV-HAP among older hospitalized patients.

LASSO and multivariate logistic regression analyses were conducted to identify the key risk factors for NV-HAP in older hospitalized patients. Consequently, CCI, NRS-2002, enteral tube feeding, Barthel Index, use of sedatives, use of NSAIDs, use of inhaled steroids, and "time at risk" were identified as the major predictors and were used to develop a predictive nomogram. The constructed nomogram showed good discrimination and strong concordance performance in predicting NV-HAP. Similarly, the results of the DCA plots revealed that the nomogram was more effective in predicting NV-HAP than intervention-all-patients or intervention-none strategies. This implied that the developed nomogram can be implemented in clinical practice.

It was also found that patients with 12 or more days of "time at risk" experienced a nearly fourfold increased risk of NV-HAP compared with those who had a "time at risk" of less than six days. A longer "time at risk" usually means a longer period of bed rest and hospitalization. Previous studies have shown that hospital stay duration is an independent predictor of healthcare-acquired infections [33, 34]. Therefore, we hypothesize that "time at risk" may help predict NV-HAP occurrence by reflecting the length of hospital stay of the patients. In addition, four medical interventions (enteral tube feeding, use of sedatives, NSAIDs, and inhaled steroids) were significantly associated with a high risk of NV-HAP in older hospitalized patients. In particular, the use of feeding tubes increased oral colonization by pathogenic organisms, causing bacteria to migrate to the lungs via the tube [7, 35]. In addition, this procedure can raise gastric volume and pressure, hence increasing the risk of gastric reflux and pulmonary aspiration [7, 35]. Several previous studies have also reported that the use of sedatives, NSAIDs, and inhaled steroids increases the risk of HAI. These drugs exert their therapeutic effects by regulating immune functions [36–39].

The Barthel Index, the CCI, and the NRS-2002 were used to assess performance in daily living activities, the burden of comorbid diseases, and nutritional status, respectively. These assessment tools were found to be independent predictors of NV-HAP. A lower Barthel Index indicates poor performance in daily activities, which may reduce respiratory secretion clearance and contribute to the development of NV-HAP [9]. Malnutrition and underlying diseases have been identified as risk factors for HAIs [40–42]. Therefore, the application of the aforementioned eight risk factors in the current model is plausible and theoretically grounded.

Currently, NV-HAP is diagnosed using imaging examinations and clinical feature changes [3, 43]. However, when symptoms appear, it is usually too late to intervene, which results in poor clinical outcomes. Using the current model, healthcare workers can implement early interventions for high-risk elderly inpatients. Interventions such as improved oral hygiene, dysphagia screening, and early mobilization should be implemented more aggressively for high-risk individuals, and this may reduce the risk of NV-HAP. Moreover, the online version of the nomogram provided a more intuitive and convenient prediction of the risk of NV-HAP disease in patients.

This study has some limitations. First, the study adopted a retrospective design and was performed in a single center, which limits the generalizability of the findings. Furthermore, although internal validation was conducted to ensure the robustness of the developed nomogram, external validation could not be conducted. Therefore, further research is needed to validate the nomogram using an external cohort. Second, the study selected a few readily available risk factors and medical interventions, leaving out others such as the risk across the continuum of care and indicators of blood gas analysis [7]. Third, although each case of NV-HAP was extensively assessed and co-confirmed by a doctor and a senior infection control practitioner to avoid misclassification bias in the current investigation, misdiagnosis and missed diagnosis may still have occurred. Finally, even though various assessment tools, such as the Barthel Index, Morse Fall Scale, and NRS-2002, were conducted by specially trained nurses, the existence of measurement bias cannot be ruled out because the screening was performed by different nurses.

## Conclusions

In conclusion, a novel nomogram combining CCI, NRS-2002, enteral tube feeding, Barthel Index, use of sedatives, use of NSAIDs, use of inhaled steroids, and "time at risk" was constructed in the present study. The nomogram showed good discrimination and calibration and hence can help healthcare workers estimate the risk of NV-HAP disease in older hospitalized patients. This will promote the implementation of early intervention for high-risk groups. However, there is a need to conduct external validation of the proposed nomogram in larger populations.

## Supplementary Information

**Additional file 1. Fig.S1.** Flow chart of the study population. **Fig. S2.** Screenshot of the online tool used for the prediction of NV-HAP risk. **Table. S1** Diagnostic efficacy of the nomogram model for estimating the risk of NV-HAP.

## Data Availability

All data generated or analysed during this study are included in this published article and Additional file 1.

## Abbreviations

HAP: Hospital-acquired pneumonia
HAIs: Healthcare-associated infections
NV-HAP: Nonventilator hospital-acquired pneumonia
VAP: Ventilator-associated pneumonia
IQR: Inter-quartile range
COPD: Chronic obstructive pulmonary disease
ICU: Intensive care unit
NSAIDs: Non-steroidal anti-inflammatory drug
CCI: Charlson comorbidity index
NRS: Nutritional risk screening
BUN: Blood urea nitrogen
ALB: Albumin
CRP: C-reaction protein
LY: Lymphocyte
WBC: White blood cell
Hb: Hemoglobin
LASSO: Least absolute shrinkage and selection operator
AIC: Akaike information criterion
DCA: Decision curve analysis
AUC: Area under curve
CI: Confidence interval
PPV: Positive predictive value
NPV: Negative predictive value
PLR: Positive likelihood ratio
NLR: Negative likelihood ratio
DOR: Diagnostic odds ratio
