# Supplementary material for: Derivation and validation of a nomogram for predicting nonventilator hospital-acquired pneumonia among older hospitalized patients
Source: BMC Pulm Med. 2022 Apr 15;22:144. doi: 10.1186/s12890-022-01941-z (PMC9011946; doi:10.1186/s12890-022-01941-z)
Supplement: Supplementary file 1 — Additional file 1. Fig.S1. Flow chart of the study population. Fig. S2. Screenshot of the online tool used for the prediction of NV-HAP risk. Table. S1 Diagnostic efficacy of the nomogram model for estimating the risk of NV-HAP. [file 12890_2022_1941_MOESM1_ESM.docx]

**Derivation and validation of a nomogram for predicting nonventilator hospital-acquired pneumonia among older hospitalized patients**

Zhihui Chen^1, 2^, Ziqin Xu^1^, Hongmei Wu^1^, Shengchun Gao^1^, Haihong Wang ^1^, Jiaru Jiang^1^, Xiuyang Li^2*^, Le Chen^1*^

Department of Infection Control, Wenzhou People's Hospital, Wenzhou, China.

Department of Epidemiology & Statistics, and Center for Clinical Big Data and Statistics, Second Affiliated Hospital, Zhejiang University College of Medicine, Hangzhou, China.

^*^Corresponding author:

Le Chen

Department of Infection Control, Wenzhou People's Hospital, 57 Canghou Road, Wenzhou, China.

E-mail: chenle1231974@126.com;

Tel: +86-13456060012

Xiuyang Li

Clinical Big Data & Statistics Center, Second Affiliated Hospital, Zhejiang University College of Medicine, 866 Yuhangtang Road, Hangzhou, 310058, China.

E-mail: lixiuyang@zju.edu.cn;

Tel: +86-571-8820-8192


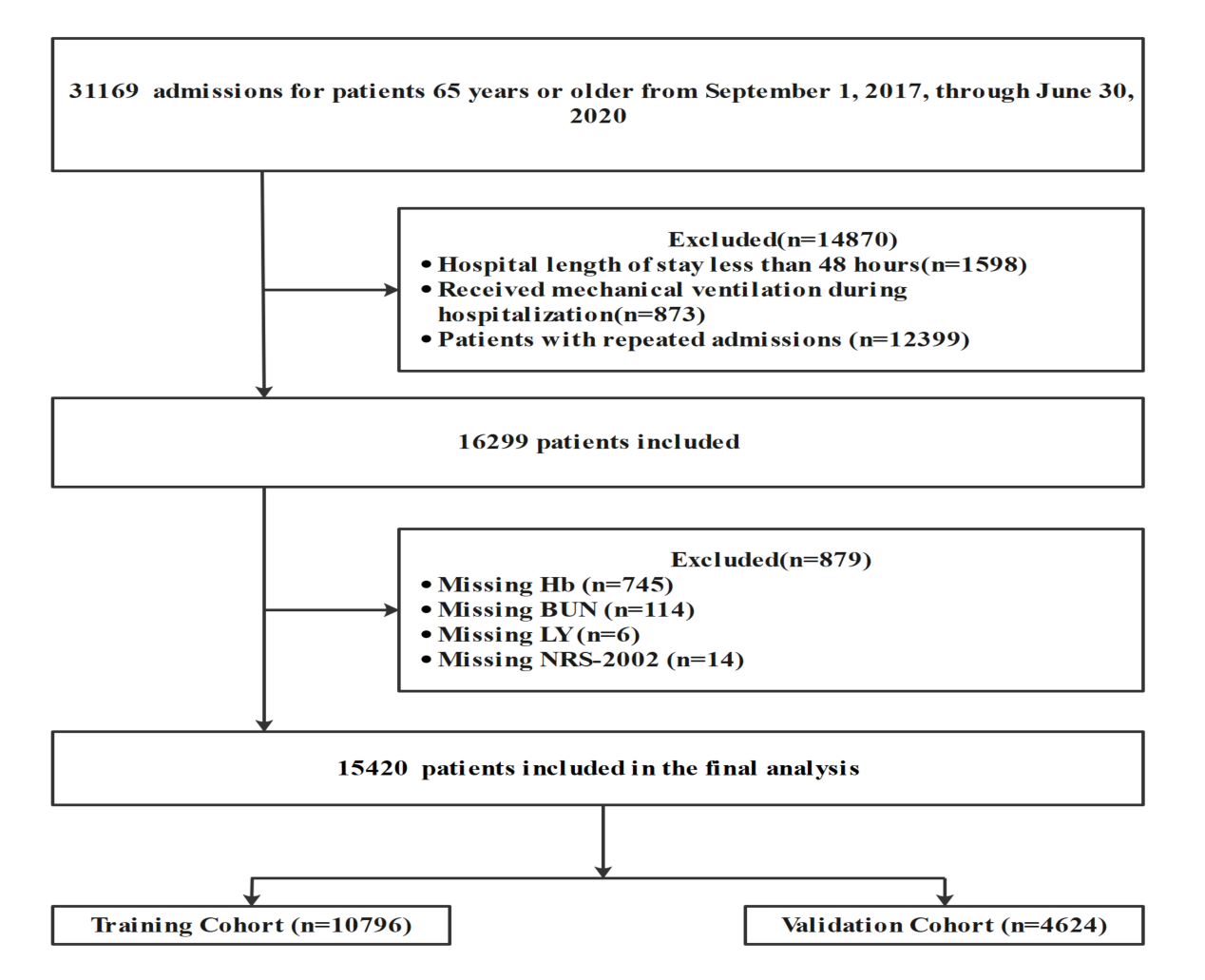


Fig.S1. Flow chart of the study population. Abbreviations: NRS, nutritional risk screening; BUN, blood urea nitrogen; LY, lymphocyte; Hb, hemoglobin.


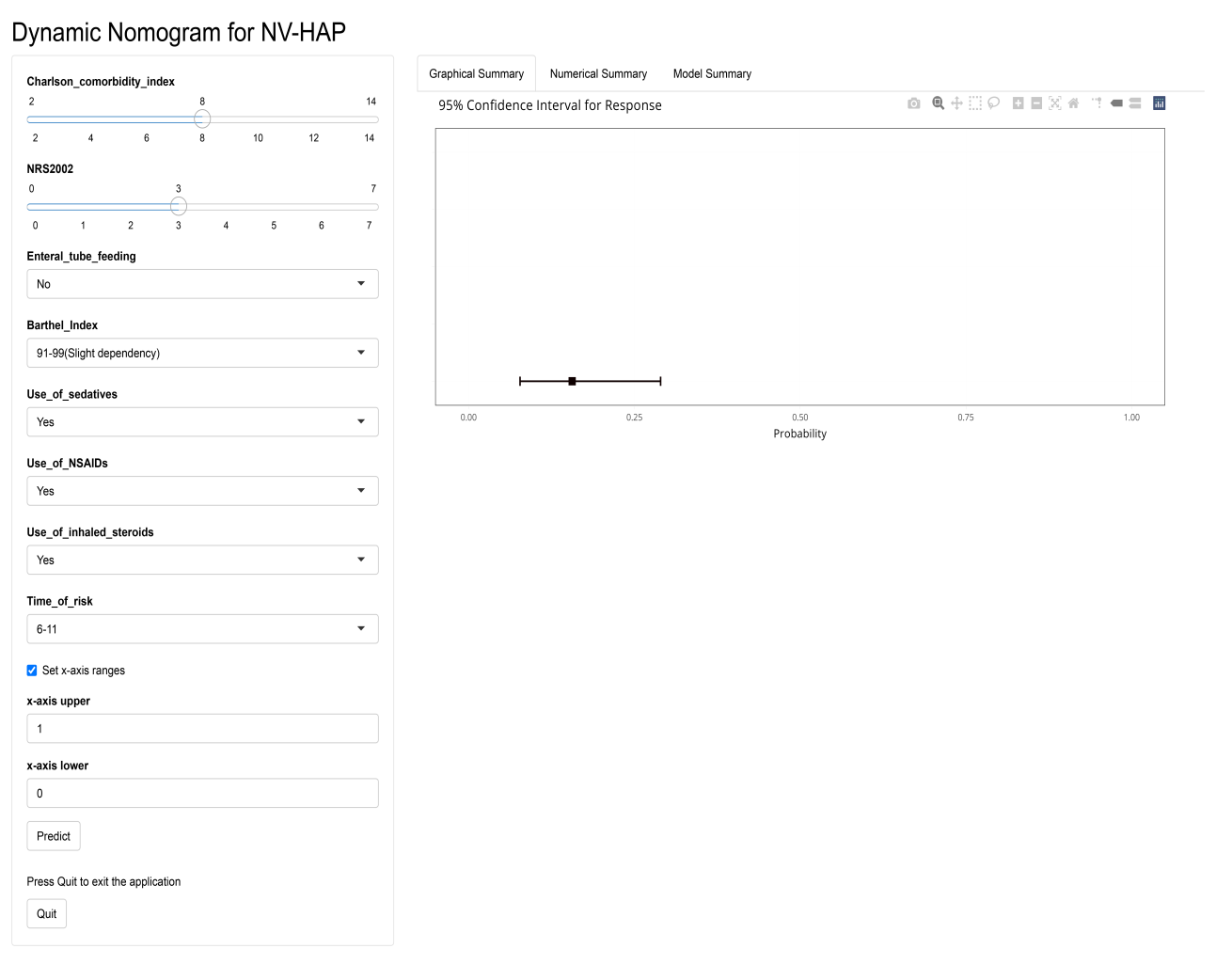


Fig.S2. Screenshot of the online tool used for the prediction of NV-HAP risk. Abbreviations: NV-HAP, nonventilator-associated hospital-acquired pneumonia; NRS, nutritional risk screening.

**Table. S1 Diagnostic efficacy of the nomogram model for estimating the risk of NV-HAP**

| Variables | Value | |
| --- | --- | --- |
|  | Training Cohort (n = 10796) | Validation Cohort (n = 4624) |
| Best threshold (%) | 1.58 | 1.74 |
| Specificity (%) | 81.94 | 82.36 |
| Sensitivity (%) | 69.93 | 70.83 |
| Accuracy (%) | 81.77 | 82.18 |
| PPV (%) | 5.27 | 5.97 |
| NPV (%) | 99.48 | 99.44 |
| PLR | 3.87 | 4.02 |
| NLR | 0.37 | 3.54 |
| DOR | 10.55 | 11.34 |

AUC, Area under curve; CI, Confidence interval; PPV, Positive predictive value; NPV, Negative predictive value; PLR, Positive likelihood ratio; NLR, Negative likelihood ratio; DOR, Diagnostic odds ratio.
